# Clinical Performance of a “No Wait” Universal Adhesive in Noncarious Cervical Lesions: A Two-year Randomized Controlled Clinical Trial

**DOI:** 10.3290/j.jad.b3240675

**Published:** 2022-08-18

**Authors:** Fatma Dilsad Oz, Meltem Nermin Dursun, Esra Ergin

**Affiliations:** a Associate Professor, Department of Restorative Dentistry, Faculty of Dentistry, Hacettepe University, Sihhiye, Ankara, Turkey. Conceptualization, data curation, formal analysis, methodology; wrote, reviewed, and edited the manuscript.; b Assistant Professor, Department of Restorative Dentistry, Faculty of Dentistry, Firat University, Elazıg, Turkey. Data curation, formal analysis.; c Professor, Department of Restorative Dentistry, Faculty of Dentistry, Hacettepe University, Sihhiye, Ankara, Turkey. Conceptualization, data curation, methodology, reviewed and edited the manuscript, supervised the study.

**Keywords:** universal adhesive, noncarious cervical lesions, self-etch, etch-and-rinse.

## Abstract

**Purpose:**

To evaluate the 24-month clinical performance of a “no wait” universal adhesive with different application modes in comparison with an etch-and-rinse and two-step self-etch adhesive in noncarious cervical lesions (NCCLs).

**Materials and Methods:**

A total of 234 noncarious cervical lesions in 34 patients were restored following 5 different adhesive approaches: 1. Clearfil Universal Bond Quick (Kuraray Noritake), self-etch mode (CUQ-SE); 2. Clearfil Universal Bond Quick, selective enamel-etch mode (CUQ-SLE); 3. Clearfil Universal Bond Quick, etch-and-rinse mode (CUQ-ER); 4. Clearfil SE Bond (Kuraray Noritake; self-etch adhesive) (CSEB); 5. Tetric N-Bond Universal (Ivoclar Vivadent), etch-and-rinse mode (TBU-ER). All NCCLs were restored with a nanohybrid composite (Tetric N-Ceram; Ivoclar Vivadent). The restorations were evaluated at baseline, 6, 12, and 24 months of clinical service regarding retention, marginal adaptation, marginal discoloration, secondary caries, post-operative sensitivity, color match, surface texture using modified United States Public Health Service (USPHS) criteria.

**Results:**

The patient recall rate at 24 months was 73.5%. Eleven restorations, 6 of the CUQ-SE group, 4 of the CSEB group and 1 of the TBU-ER group, were clinically unacceptable due to retention loss. Regarding marginal adaptation and discoloration, CUQ-SE and CSEB groups exhibited higher bravo scores than other groups after 24 months (p < 0.05). At the end of 24-month examinations, no significant differences were detected among the groups regarding secondary caries, post-operative sensitivity, color match and surface texture.

**Conclusion:**

The clinical survival rates of the “no wait” universal adhesive at self-etch mode after 24 months were not acceptable. The “no wait” universal adhesive showed clinically acceptable performance in selective enamel-etch and etch-and-rinse mode, according to the evaluated USPHS criteria.

Dental adhesives have been simplified over time to become more user friendly, thanks to fast application and the availability of less technique-sensitive products available. Phosphoric acid etching remains the gold standard for enamel bonding. Due to their weak and insufficient etching potential on enamel, self-etch adhesives are not able to form a reliable bond and present with lower bond strengths than etch-and-rinse adhesives.^47^ Etching leads to increased collagen degradation by activating metalloproteinases in dentin. Nevertheless, self-etch adhesives can be useful to decrease the effects of degradation.^43^ Self-etch adhesives are classified into two types: two-step and one-step self-etch adhesives. Both types require a waiting period before light curing; however, the waiting period and application method differ according to the brand. In two-step self-etch adhesives, the active ingredients are separated, whereas in one-step adhesives, the conditioning, priming, and application of the adhesive resin are combined. One-step self-etch adhesives are more hydrophilic because the acidic monomer concentration is increased. As a result, a compromised adhesive-dentin bond with semi-permeable hybridization is formed. This semi-permeable layer leads to premature degradation of adhesive-dentin bonds.^42^ Self-etch adhesives contain acidic monomers that simultaneously condition and prime the dental substrate; they have a shorter application time and are less technique sensitive, thus promoting reliable clinical performance.^48^ Also, they result in a lower incidence of post-operative sensitivity than do etch-and-rinse adhesives.^48^ On the other hand, the main problem with self-etch adhesives is that their bond to enamel is less durable than that of etch-and-rinse adhesives.^48^

Multi-mode universal adhesives with fewer steps have been developed as viable options for adhesion to enamel and dentin.^9^ Self-etch, selective enamel-etch and etch-and-rinse adhesion strategies can all be used with universal adhesives and they are also claimed to be able to substitute for silane agents when bonding ceramic materials and metals.^23^ The definition of a universal adhesive in the literature describes it as a single-bottle, no-mix adhesive that performs with any bonding strategy and adheres adequately to tooth structure as well as to different restorative materials.^2,22^ A clinical study reported that self-etch and selective enamel-etch techniques with a universal adhesive produced clinically acceptable results in resin composite restorations of noncarious cervical lesions (NCCLs) after 2 years.^33^ The literature mentions that prolonged application times of adhesives can increase the adhesive-dentin microtensile bond strength^7^ and make the adhesive layer more stable over time.^31^ Longer application times are expected to increase monomer infiltration.^7,36^ On the other hand, prolonged application times are not considered to be practical in clinical situations, and reduced application times are desired by dentists.

Recently, some universal adhesives were tested with different application options to determine whether their clinical application could be accelerated. Microtensile bond strengths were evaluated immediately after applying the bonding agent with “no wait” timing and compared with those after leaving the bonding agent undisturbed for 10 s.^36^ The shortened application time was reported to result in insufficient bonding and lower bond strength for Clearfil Universal Bond (Kuraray Noritake; Tokyo, Japan) and G-Premio Bond (GC; Tokyo, Japan); however, it did not affect Single Bond Universal (3M Oral Care; St Paul, MN, USA).^36^ Another in-vitro investigation also reported that a universal adhesive (G-Premio Bond GC) had lower bonding performance without a waiting period compared to leaving the adhesive undisturbed for 10 s.^16^

NCCLs are characterized by the loss of cervical hard dental tissue at the level of the cementoenamel junction.^40^ Some NCCLs are superficial, whereas others are more profound defects, and they may have different forms and dimensions.^40^ These characteristics influence the choice of NCCL treatment and the longevity of restoration efforts.^37^ NCCLs should be restored to prevent further hard tissue loss and improve esthetics. Dentin hypersensitivity is also a reason to treat NCCLs with the placement of an adhesive restoration.^26^ However, restorative procedures are challenging, because NCCLs have non-retentive shapes, and in some cases, they have sclerotic dentin and cement margins that are unfavorable for adhesion.^37^ A recent meta-analysis reported that bonding strategies do not affect the clinical performance of adhesives used to restore NCCLs.^13^ In contrast, a different meta-analysis mentioned that for the restoration of NCCLs, clinicians might consider applying universal adhesives in selective enamel-etching mode, since it could lead to more predictable retention compared to the self-etch approach.^17^

Adhesives have improved in recent years, and today, their efficacy in treating NCCLs is clearly better. The selective enamel-etching approach has become more popular due to reports of improved clinical performance with less hypersensitivity with this application method.^6^ In the literature, clinical success has been obtained in NCCLs with some two-step self-etch adhesives, such as Clearfil SE Bond (Kuraray Noritake).^51^ A 13-year clinical trial conducted with a two-step self-etch adhesive (Clearfil SE Bond) found that significantly more marginal adaptation problems occurred on the enamel margins in the non-etched group compared to the etched group.^27^ In addition, clinical studies have demonstrated that although universal adhesives exhibit acceptable short-term performance,^20,25^ there was some marginal degradation 3 years after treating NCCLs using a self-etch strategy.^19^ Two recent studies which evaluated different adhesive strategies of universal adhesives found that the etch-and-rinse strategy performed better than did the self-etch strategy after three^24^ or five years.^12^ In addition, some laboratory studies found that over time, the marginal integrity of the bonding agent to enamel deteriorated.^4,32^

Recently, a universal adhesive with no waiting time was introduced to the market. An in-vitro study evaluated the microtensile bond strength of this “no wait” universal adhesive (Clearfil Universal Bond Quick, Kuraray Noritake) and reported that its performance was similar to that of a universal adhesive applied for 20 s.^1^ Also, Clearfil SE Bond exhibited superior microtensile bond strengths vs Clearfil Universal Bond Quick.^1^

The purpose of this randomized, controlled clinical trial was to evaluate the clinical performance of the “no wait” universal adhesive (Clearfil Universal Bond Quick, Kuraray Noritake) using three application strategies together with a two-step self-etch adhesive and an etch-and-rinse adhesive in restoring NCCLs after 24 months. The null hypothesis was that there would be no differences between study groups regarding retention, marginal discoloration, marginal adaptation, postoperative sensitivity, and secondary caries based on the USPHS criteria.

## MATERIALS AND METHODS

### Ethical Approval and Protocol Registration

This randomized controlled clinical study was performed following the CONSORT statement and is registered at ClinicalTrials.gov (Clinical Trials Number: NCT04481087). The protocol was approved by the Institutional Research Ethics Committee for Clinical Investigations (KA-19012). All participants were informed about the content of the study; signed, written consent forms were completed prior to the treatments.

### Sample Size Calculation

Power analysis using G*Power statistical software (version 3.1) was used to calculate the minimum sample size to achieve an effect difference between the groups of 90% power (w=0.30). However, the minimum sample size was increased to 40 restorations per group in order to compensate potential loss and drop-outs. Since all eligible NCCLs of the participants who met the inclusion criteria were included in the study, the number of restorations in the groups were 46-47 at the beginning of the study.

### Study Design and Patient Selection

A clinician recruited participants who met the inclusion criteria (Table 1) from among patients seeking routine dental care at the Restorative Dentistry Department, Hacettepe University. A total of 59 patients were examined by one clinician to determine whether they met the eligibility criteria. The cervico-incisal or cervico-occlusal height of the lesions was measured using a periodontal probe. Non-retentive lesions with a cavosurface margin involving at most 50% of the enamel were included. One clinician carried out assessments using an explorer, a mouth mirror, and a periodontal probe (Table 2).

**Table 1:** Inclusion and exclusion criteria for participants

Inclusion criteria	Exclusion criteria
18 years or older At least 20 teeth under occlusion At least five noncarious cervical lesions (NCCLs) that needed restoration in different teeth and that were similar in size (depth), ranging from 1 to 3 mm	Uncontrolled caries Xerostomia Medical problems preventing patients from attending review visits Poor gingival health Bruxism Removable partial dentures Patients with severe hypersensitivity (checked with a cold test)


**Table 2:** Characteristics of NCCLs included in the study

	Number of NCCLs (n)
Shape (degree of angle)	
< 45	
45–90	56
90–135	141
> 135	37
Cervico-incisal height (mm)	
1.5–2.5	8
> 2.5-4.0	170
> 4.0	56
	

### Randomization

Randomization was carried out using the “Research Randomized Program” (http://www.randomizer.org/form.htm). A clinician who was not involved in the research protocol carried out this process. Teeth were randomly assigned to five groups (CUQ-SE: Clearfil Universal Bond Quick [Kuraray Noritake], self-etch mode; CUQ-SLE: Clearfil Universal Bond Quick, selective enamel-etch mode; CUQ-ER: Clearfil Universal Bond Quick, etch-and-rinse mode; CSEB: Clearfil SE Bond [Kuraray Noritake]; TBU-ER: Tetric N-Bond Universal [Ivoclar Vivadent], etch-and-rinse mode) using a table of random numbers. Each patient received at least five restorations. If the patient had more included lesions, the same randomization protocol was followed for subsequent lesions.

### Restorative Procedures

In this clinical trial, 34 patients were enrolled (mean age: 49 years, 12 males and 22 females) (Table 3). All lesions were cleaned with pumice using a rotating rubber cup in a slow-speed handpiece, washed, and dried but not desiccated before restoration. In each patient, five or more NCCLs were restored randomly following five protocols established according to the manufacturers’ recommendations (Table 4). All lesions were isolated using cotton rolls, suction, and a gingival retraction cord (Ultrapak, Ultradent; South Jordan, UT, USA) before treatment. Lesions in the CUQ-SE (n = 46), CUQ-SLE (n = 47), and CUQ-ER (n = 47) groups were treated with Clearfil Universal Bond Quick (Kuraray Noritake) using self-etch, selective enamel-etch, and etch-and-rinse strategies, respectively, those in the CSEB group (n = 47) were treated with Clearfil SE Bond (Kuraray Noritake) (self-etch), and those in the TBU-ER group (n = 47) were treated with Tetric N-Bond Universal (Ivoclar Vivadent) using an etch-and-rinse strategy.

**Table 3:** Distribution of participants and NCCLs according to gender and age

Characteristics of research subjects	Number of patients	Number of NCCLs (n %)
**Gender distribution**		
Male	12	83 (35.3)
Female	22	151 (64.7)
**Age distribution (years)**		
20–29	0	-
30–39	4	30 (12.8)
40–49	17	112 (47.8)
50–59	10	71 (30.3)
60–65	3	21 (9.1)


Shade selection was carried out before lesion restoration. All lesions were restored with a resin composite (Tetric N-Ceram, Ivoclar Vivadent) according to the manufacturer’s recommendations, including light curing for 20 s (Table 4).

**Table 4:** Materials used in the study

Material / Manufacturer	Composition	Applications
Tetric N-Ceram / Ivoclar Vivadent; Schaan, Liechtenstein	Bisphenol A diglycidylmethacrylate (bis-GMA), urethanedimethacrylate (UDMA), ethyoxylated bisphenol A dimethacrylate, ytterbium trifluoride	The composite was placed in the lesions and light cured for 20 s.
Clearfil Universal Bond Quick / Kuraray Noritake; Tokyo, Japan	Bis-GMA, ethanol, 2-hydroxyethyl methacrylate, 10-methacryloyloxydecyl dihydrogen phosphate, hydrophilic amide monomers, colloidal silica, silane coupling agent, sodium fluoride, dl-camphorquinone, water	Self-etch mode: The bonding agent was applied with an applicator brush to the entire lesion using a rubbing motion (no waiting time). The adhesive was dried gently with oil-free air for 5 s and then light cured with an LED-curing unit (Radii Plus, SDI; Bayswater, Victoria, Australia) for 10 s. Selective enamel-etch mode: Enamel was etched with 37% phosphoric acid (Total Etch, Ivoclar Vivadent; Schaan, Liechtenstein) for 10 s. The bonding agent was applied with an applicator brush to the entire lesion using a rubbing motion (no waiting time). The adhesive was dried gently with oil-free air for 5 s and then light cured with an LED-curing unit (Radii Plus; SDI) for 10 s. Etch-and-rinse mode: Enamel and dentin were etched with 37% phosphoric acid (Total Etch, Ivoclar Vivadent) for 10 s and the bonding agent was applied with an applicator brush to the entire lesion using a rubbing motion (no waiting time). The adhesive was dried gently with oil-free air for 5 s and then light cured with an LED-curing unit (Radii Plus; SDI) for 10 s.
Clearfil SE Bond / Kuraray Noritake	**Primer** 10-methacryloyloxydodecyl dihydrogen phosphate (MDP), 2-hydroxyethyl methacrylate (HEMA), hydrophilic aliphatic dimethacrylate, dl-camphorquinone, N,N-diethanol-p-toluidine, water **Bonding agent** 10-methacryloyloxydodecyl dihydrogen phosphate (MDP), bisphenol A diglycidyl methacrylate (bis-GMA), 2-hydroxyethyl methacrylate (HEMA), hydrophobic aliphatic dimethacrylate, dl-camphorquinone, N,N-diethanol-p-toluidine, colloidal silica	The primer was applied with an applicator brush to the entire lesion and left undisturbed for 10 s. Then, the primer was dried with a mild air flow. Afterwards, the bonding agent was applied with an applicator brush to the entire lesion and dried with a mild air flow. Finally, the bonding agent was light cured with an LED-curing unit (Radii Plus; SDI) for 10 s.
Tetric N-Bond Universal / Ivoclar Vivadent	10-methacryloyloxydodecyl dihydrogen phosphate (MDP), bisphenol A diglycidyl methacrylate (bis-GMA), 2-hydroxyethyl methacrylate (HEMA), dodecanediol dimethacrylate (D3MA), ethanol, water, highly dispersed silicon dioxide, initiators, stabilizers	Etch-and-rinse mode: Enamel was etched for 30 s and dentin was etched for 15 s using 37% phosphoric acid (Total Etch, Ivoclar Vivadent). The bonding agent was applied with an applicator brush to the entire lesion and agitated for 20 s. Compressed air was applied until the bonding agent appeared immobile. Then, light curing was performed with an LED-curing unit (Radii Plus; SDI) for 10 s.


Flame-shaped fine finishing diamond burs were used for contouring with a high-speed handpiece under water spray. Then, finishing and polishing disks (OptiDisc, Kerr; Orange, CA, USA) were used. The extra-coarse (dark-brown) disk was used on the restoration sites where contouring and rough finishing was required. The coarse/medium (light-brown), the fine (orange) and the extra-fine (yellow) disk were used for all restorations in sequence.

### Clinical Evaluation

The restorations were evaluated at baseline and at 6, 12, and 24 months based on all mentioned USPHS criteria by two experienced examiners. The examiner scoring was previously calibrated. The examiners were blinded to the restorative procedures and had not placed the restorations. Calibration was performed with 10 patients selected by an examiner who was not involved in the study. First, photographs were taken by this examiner. Afterwards, the patients were examined clinically by two experienced examiners who were involved in the study and scored the restorations for all criteria. The evaluators also checked their clinical decisions on magnified (10X) intraoral photographs. Intra- and inter-examiner agreement scores of at least 85% were necessary before beginning the evaluation.

The variables retention, marginal adaptation, marginal discoloration, postoperative sensitivity, and secondary caries were evaluated. Scores were assigned as follows: alpha: clinically very good; bravo: clinically sufficient/satisfactory; and charlie: clinically poor. The examiners evaluated all restorations independently. In case of disagreement, the examiners had to reach a consensus before the participant was dismissed.

### Statistical Analysis

Descriptive statistics are used to present the frequency distributions of the evaluated criteria. The chi-squared test was used to compare the groups at baseline and at 6, 12, and 24 months. To distinguish differences in marginal adaptation and marginal discoloration scores within each group over time, further analyses were carried out using Cochran’s Q test followed by McNemar’s test to compare the data obtained at each evaluation period with baseline data. The survival rate of the restorations over time was calculated using the Kaplan-Meier analysis. The log-rank test was used to compare the survival distribution of restorations. For all analyses, statistical significance was set at p < 0.05.

## RESULTS

**Fig 1:**
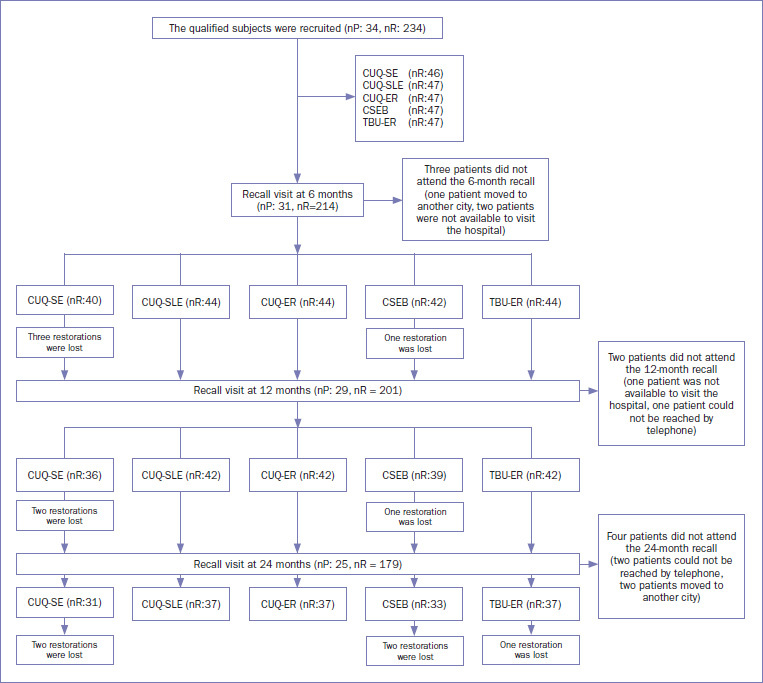
Flow diagram of the study. CUQ-SE: Clearfil Universal Bond Quick, self-etch mode; CUQ-SLE: Clearfil Universal Bond Quick, selective etch mode; CUQ-ER: Clearfil Universal Bond Quick, etch-and-rinse mode; CSEB: Clearfil SE Bond; TBU-ER: Tetric N-Bond Universal, etch-and-rinse mode.

A flow chart of the study is shown in Fig 1. In this study, 234 restorations were performed in 34 patients using different adhesives and strategies. The distribution of NCCLs according to tooth type, arch position, and adhesive/strategy used is given in Table 5. Most restorations were performed on premolars (61.1%). For the other restorations, 11.9% were performed on incisors, 18.8% were performed on canines, and 8.1% were performed on molars. Recall rates were 91.4%, 85.2%, and 73.5% at the 6-, 12- , and 24-month evaluations, respectively. Clinical assessment outcomes are given in Table 6.

**Table 5:** Distribution of NCCLs according to tooth type and arch

Number of NCCLs	CUQ-SE	CUQ-SLE	CUQ-ER	CSEB	TBU-ER	Total (n %)
**Arch distribution**						
Maxillary	36	25	27	29	39	156 (66.7)
Mandibular	10	22	20	18	8	78 (33.3)
**Tooth distribution**						
Incisors	5	13	3	1	6	28 (11.9)
Canines	6	2	6	16	14	44 (18.8)
Premolars	31	31	33	29	19	143 (61.1)
Molars	4	1	5	1	8	19 (8.1)

CUQ-SE: Clearfil Universal Bond Quick (Kuraray Noritake), self-etch mode; CUQ-SLE: Clearfil Universal Bond Quick, selective etch mode; CUQ-ER: Clearfil Universal Bond Quick, etch-and-rinse mode; CSEB: Clearfil SE Bond (Kuraray Noritake); TBU-ER: Tetric N-Bond Universal (Ivoclar Vivadent), etch-and-rinse mode.

**Table 6:** Clinical evaluation outcomes of different adhesive systems/strategies

Evaluation criteria	Score	Baseline n (%)					6-month n (%)					12-month n (%)					24-month n (%)				
		CUQ-SE (n = 46)	CUQ-SLE (n = 47)	CUQ-ER (n = 47)	CSEB (n = 47)	TBU-ER (n = 47)	CUQ-SE (n = 40)	CUQ-SLE (n = 44)	CUQ-ER (n = 44)	CSEB (n = 42)	TBU-ER (n = 44)	CUQ-SE (n = 36)	CUQ-SLE (n = 42)	CUQ-ER (n = 42)	CSEB (n = 39)	TBU-ER (n = 42)	CUQ-SE (n = 31)	CUQ-SLE (n = 37)	CUQ-ER (n = 37)	CSEB (n = 33)	TBU-ER (n = 37)
Retention	Alpha	46 (100)	47 (100)	47 (100)	47 (100)	47 (100)	40 (93)	44 (100)	44 (100)	42 (97.7)	44 (100)	36 (94.7)	42 (100)	42 (100)	39 (97.5)	42 (100)	31 (96.9)	37 (100)	37 (100)	33 (94.3)	37 (97.4)
	Bravo																				
	Charlie						3 (7)			1 (2.3)		2 (5.3)			1 (2.5)		1 (3.1)			2 (5.7)	1 (2.6)
Marginal adaptation	Alpha	46 (100)	47 (100)	47 (100)	47 (100)	47 (100)	33 (82.5)	44 (100)	43 (97.7)	41 (97.6)	44 (100)	26 (72.2)	40 (95.2)	41 (97.6)	32 (82.1)	41 (97.6)	23 (74.2)	34 (91.9)	34 (75.8)	25 (75.8)	35 (94.2)
	Bravo						7 (17.5)		1 (2.3)	1 (2.4)		10* (27.8)	2 (4.8)	1 (2.4)	7* (17.9)	1 (2.4)	8* (25.8)	3 (8.1)	3 (24.2)	8* (24.2)	2 (5.4)
	Charlie																				
Marginal discoloration	Alpha	46 (100)	47 (100)	47 (100)	47 (100)	47 (100)	39 (97.5)	44 (100)	44 (100)	42 (100)	44 (100)	31 (86.1)	42	42	38 (97.4)	42	25 (80.6)	36 (97.3)	36 (97.3)	28 (84.8)	37
	Bravo						1 (2.5)					5 (13.9)			1 (2.6)		6* (19.4)	1 (2.7)	1 (2.7)	5* (15.2)	
	Charlie																				
Post-operative sensitivity	Alpha	46 (100)	47 (100)	47 (100)	47 (100)	47 (100)	40 (100)	44 (100)	44 (100)	42 (100)	44 (100)	36 (100)	42 (100)	42 (100)	39 (100)	42 (100)	31 (100)	37 (100)	37 (100)	33 (100)	37 (100)
	Bravo																				
	Charlie																				
Secondary caries	Alpha	46 (100)	47 (100)	47 (100)	47 (100)	47 (100)	40 (100)	44 (100)	44 (100)	42 (100)	44 (100)	36 (100)	42 (100)	42 (100)	39 (100)	42 (100)	31 (100)	37 (100)	37 (100)	33 (100)	37 (100)
	Bravo																				
	Charlie																				
Color match	Alpha	46 (100)	47 (100)	47 (100)	47 (100)	47 (100)	40 (100)	44 (100)	44 (100)	42 (100)	44 (100)	36 (100)	42 (100)	42 (100)	39 (100)	42 (100)	31 (100)	37 (100)	37 (100)	33 (100)	37 (100)
	Bravo																				
	Charlie																				
Surface texture	Alpha	46 (100)	47 (100)	47 (100)	47 (100)	47 (100)	40 (100)	44 (100)	44 (100)	42 (100)	44 (100)	36 (100)	42 (100)	42 (100)	39 (100)	42 (100)	30 (96.8)	37 (100)	35 (94.6)	33 (100)	37 (100)
	Bravo																1 (3.2)		2 (5.4)		
	Charlie																				

* Indicates significant difference within each group when compared to baseline (p < 0.05). CUQ-SE: Clearfil Universal Bond Quick, self-etch mode; CUQ-SLE: Clearfil Universal Bond Quick, selective enamel-etch mode; CUQ-ER: Clearfil Universal Bond Quick, etch-and-rinse mode; CSEB: Clearfil SE Bond, self-etch mode TBU-ER: Tetric N-Bond Universal, etch-and-rinse mode.

**Fig 2:**
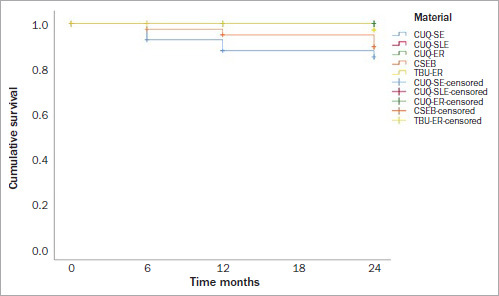
Survival curves for the 5 tested groups (CUQ-SE [Clearfil Universal Bond Quick in self-etch mode], CUQ-SLE [Clearfil Universal Bond Quick in selective enamel-etch mode], CUQ-ER [Clearfil Universal Bond Quick in etch-and-rinse mode], CSEB [Clearfil SE Bond, TBU-ER [Tetric N-Bond Universal in etch-and-rinse mode]).

The retention rate was 100% for the CUQ-SLE, CUQ-ER, and TBU-ER groups at the 6-month evaluation. Three (7%) restorations in the CUQ-SE group and 1 (2.3%) in the CSEB group were lost after 6 months. At the 12-month evaluation, 2 (5.3%) restorations in the CUQ-SE group and 1 (2.5%) restoration in the CSEB group were lost. After 24 months, 1 (3.1%) restoration in the CUQ-SE group, 2 (5.7%) restorations in the CSEB group, and 1 (2.6%) restoration in the TBU-ER group were lost. The Kaplan-Meier analysis showed significant differences between the survival rates of the test groups after 24 months (Fig 2). The 24-month survival rates in the CUQ-SE, CUQ-SLE, CUQ-ER, CSEB, and TBU-ER groups were 87%, 100%, 100%, 91.5%, and 95.3%, respectively. Lower survival rates were observed in the CUQ-SE and CSEB groups after 24 months (log-rank test: p < 0.05).

At the 6-month assessment, 7 (17.5%) restorations in the CUQ-SE group, 1 (2.3%) in the CUQ-ER group, and 1 (2.4%) in the CSEB group scored bravo for marginal adaptation. Restorations in the CUQ-SE group were scored as bravo significantly more often than those in the other groups (p = 0.001). After 12 months, 10 (27.8%) restorations in the CUQ-SE group and 7 (17.9%) in the CSEB group scored bravo, significantly more than those in the other groups (p = 0.001). After 24 months, groups CUQ-SE (25.8%) and CSEB (24.2%) were again rated bravo for marginal adaptation significantly more often than the CUQ-SLE, CUQ-ER and TBU-ER groups (p = 0.03).

Regarding marginal discoloration, 5 (13.9%) restorations in the CUQ-SE group and 1 in the CSEB group were given bravo scores after 12 months. The CUQ-SE group revealed a significantly higher rate of bravo scores than did the other experimental groups (p = 0.001). After 24 months, 6 (19.4%) restorations in the CUQ-SE group, 5 (15.2%) in the CSEB group, 1 in the CUQ-SLE group, and 1 in the CUQ-ER group were given bravo scores. The restorations in CUQ-SE and CSEB groups had a significantly higher rate of bravo scores than the restorations in other groups (p = 0.003).

No significant differences were found between the tested groups for secondary caries, post-operative sensitivity, color matching, or surface texture using modified USPHS criteria (p > 0.05).

Cochran’s Q test showed a significant change in marginal adaptation in the CUQ-SE and CSEB groups at the 12- (CUQ-SE, p = 0.001; CSEB, p = 0.03) and 24-month (CUQ-SE, p = 0.001; CSEB, p = 0.002) examinations. In addition, significant changes in marginal discoloration were observed in the CUQ-SE group after 24 months (p = 0.01).

## DISCUSSION

Several contemporary universal adhesives have been documented to provide adequate bonding performance.^12,34,49^ However, the clinical longevity of the bonded restorations still depends on the application strategy and degradation of the adhesive-tooth interface. Ensuring effective adhesion between two different hard tissues is a challenge for adhesives.

For clinicians, a shorter application time is appealing; however, simplification may cause negative consequences such as unsatisfactory infiltration of adhesives and problems with solvent evaporation, which may result in impaired adhesion. To address this, after developing one-step adhesives, manufacturers started to work on reducing and even eliminating waiting periods during the application of adhesive resins without compromising effective bonding. Currently, there are several types of adhesives on the market with varying application procedures and different waiting periods. Currently, controversy exists over the findings of published studies. An in-vitro study reported that doubling the application time of the acetone-based self-etch adhesive Futurabond (Voco) increased the dentin bond strength of this material.^44^

On the other hand, some published studies showed the results of universal adhesives with shortened application times. Saikaew et al^36^ demonstrated that shortening the application time of the acetone-based universal G-Premio Bond (GC) resulted in insufficient solvent evaporation and lower microtensile bond strength. In contrast, the ethanol-based Clearfil Universal Bond (Kuraray Noritake) was reported to exhibit similar bond strength when a shortened application time was used vs the manufacturer’s recommended application time on bur-cut enamel.^12^ Similarly, another in-vitro study conducted with Clearfil Universal Bond Quick reported that there was no significant difference in microtensile bond strength between a “no wait” application procedure vs a waiting period of 10 s.^38^ Although it is impossible to compare the findings of the present clinical study with the aforementioned in-vitro results due to the dissimilar nature of the study designs, the current findings indicate that there are no significant differences in adhesive performance of the tested “no wait” universal adhesive using an etch-and-rinse or selective enamel-etching strategy or compared to the positive control (an etch-and-rinse adhesive) over 24 months.

Adhesive monomers influence the bond strength and durability of materials. Having been shown to exhibit polar behavior, which promotes adhesion and the protection of collagen fibers through the formation of methacryloyloxydodecyl dihydrogen phosphate (MDP)-calcium salts, 10-MDP is currently one of the most reliable monomers available on the market.^8^ 10-MDP monomers can bond to calcium, build cross-linked complexes with collagen fibers in the hybrid layer, and produce an acid-base-resistant zone at the adhesive interface, which increases resistance to biodegradation of the hybrid layer.^34^ Loguercio et al^19^ and Perdigao et al^24^ reported that adhesives containing 10-MDP monomers have shown high clinical success rates after 3 years. Therefore, all adhesives used in the present study included 10-MDP-monomer-containing materials to eliminate confounding factors related to the use of different monomers and ascertain the influence of the different adhesive strategies employed. In the present study, the 10-MDP-containing universal adhesive used in etch-and-rinse mode produced better results.

NCCLs are the best choice for testing the clinical performance of adhesives. Both enamel and dentin bonding can be evaluated with these lesions, with minimal retention needed to assess bonding performance.^30^ The most frequently tested self-etch adhesive, Clearfil SE Bond (Kuraray Noritake), had the lowest annual failure rate in a systematic review, and its high bonding quality was attributed to the presence of 10-MDP, which helps the adhesive to form a stable chemical bond.^50^ In particular, the strong hydrophobic nature of the structure protects the hybrid layer against degradation.^4^ Two long-term clinical trials reported that Clearfil SE Bond had a 97% survival rate in NCCL restorations.^28,45^ However, in this clinical study, restorations using Clearfil SE Bond had a 91.5% survival rate after 2 years. Another clinical trial showed a 3.9% retention loss for a self-etch adhesive and a 2.2% retention loss for an etch-and-rinse adhesive at 6 months.^46^ Similar to those findings, in the present study, all self-etch adhesives applied had failure rates lower than 5% after 6 months, and the adhesives applied using the etch-and-rinse strategy (Clearfil Universal Bond Quick and Tetric N-Bond Universal) exhibited the lowest retention-loss rates after 24 months. Several clinical studies of NCCLs indicated that applying adhesives with an etching step resulted in superior clinical performance compared to self-etch adhesives, as the bonding interface is protected against degradation.^18,48^ A systematic review reported that the annual average failure rates of three-step etch-and-rinse and two-step self-etch adhesives were 4.8% and 4.7%, respectively.^29^ These adhesives had the highest adhesion rates for NCCL restoration. In addition, it was pointed out that the highest annual failure rate (8.1%) was observed with simplified one-step self-etch adhesives. Any simplification in the application of adhesives results in the loss of bonding effectiveness in clinical practice due to the evaporation of interface components.^11^ In the present study, use of the “no wait” universal adhesive, which has the simplest application method, resulted in the lowest survival rate. Also, marginal adaptation in this group worsened significantly over time.

The newly developed multimode universal adhesives are reported to have higher bond strength when applied using an etch-and-rinse mode instead of a self-etch mode in laboratory studies.^22^ However, Wagner et al^49^ found that the addition of an etching step did not significantly affect the bond strength of universal adhesives. Some clinical trials compared different application modes for universal adhesives and demonstrated that the application mode did not affect the clinical survival rates.^19,20^ However, Loguercio et al^19^ found that the self-etch strategy was associated with worse marginal adaptation after 3 years. Haak et al^15^ clinically evaluated a universal adhesive using optical coherence tomography images, and reported that small fractures occurred three times more often in restorations bonded using a self-etch strategy compared to a selective enamel-etch strategy. Another recently published study testing a dual-cure universal adhesive mentioned that, although restorations applied with the self-etch strategy exhibited more marginal discrepancies, the application mode employed did not affect the restoration retention rate.^10^ Similar to the present study, Ruschel et al^35^ reported that when a universal adhesive was applied using the self-etch strategy, marginal degradation (20%) developed after 3 years. In a systematic review, it was pointed out that selective enamel etching prior to the application of self-etch adhesives to NCCLs resulted in restorations with greater longevity.^41^ In the present study, restorations placed using the “no wait” universal adhesive were associated with a significantly lower survival rate compared to those using the selective enamel-etch strategy. Thus, the null hypothesis was rejected.

The pH of each adhesive may also influence bonding durability. Looking at the adhesive materials in this study, the pH of Clearfil Universal Bond Quick is 2.3 and that of the self-etch adhesive Clearfil SE Bond is 2.0, which are both considered mildly acidic. On the other hand, Tetric N-Bond Universal has a pH of approximately 2.5–3.0, which is classified as ultra-mild.^21^ In the present study, Clearfil Universal Bond Quick and Tetric N-Bond Universal produced similar clinical results with an etch-and-rinse strategy; therefore, the pH did not appear to cause a clinically significant difference. Correspondingly, Söderholm et al^39^ mentioned that the pH did not affect the clinical outcomes of the adhesives used.

According to the present findings, the CUQ-SE and CSEB groups received significantly more bravo scores for marginal adaptation and discoloration than did the other groups after 24 months. A systematic review reported that eight randomized clinical trials which evaluated composite restorations for NCCLs found no differences in marginal adaptation, marginal discoloration, post-operative sensitivity, or secondary caries when universal adhesives were used with an etch-and-rinse or self-etch strategy.^3^ However, the etch-and-rinse strategy resulted in a higher retention rate and a lower incidence of restoration fractures.^3^ These results are in agreement with the outcomes of this clinical investigation. Marginal discoloration was detected over time in some clinical studies, but this could usually be removed by repolishing.^5^ Loguercio et al^19^ compared different universal adhesive application strategies and reported that when the universal adhesive was applied using a self-etch strategy, significant signs of degradation related to marginal adaptation and marginal discoloration were seen at 36 months. Within the 24-month evaluation period in the current study, minor deterioration was observed in terms of marginal adaptation and discoloration in the groups in which the self-etch strategy was used for both adhesive brands tested. It can be speculated that enamel etching may still be more important than the material type, application time, or acidity of the adhesive. However, reducing the application time and the usage of simpler adhesives is important in many ways. A quick application would reduce the possibility of contamination and unpleasant experience during the acid etching process, while increasing patient satisfaction, cooperation, and comfort. In addition, by using time-saving adhesives, the cavities could be restored more easily and quickly, especially when treating patients with special needs, eg, children, elderly and disabled patients, where the dentist is under greater pressure to work quickly.

A study reported that sclerotic lesions did not show significantly different retention rates compared to non-sclerotic lesions.^45^ Also, Ruscel et al^34^ showed that restorations were more likely to present marginal discoloration in teeth with greater levels of dentin sclerosis; however, the comparison was not statistically significant.

As in all studies, this clinical trial has some limitations, chiefly the exclusion of patients with bruxism and xerostomia, which are frequently present in patients with NCCLs. Although this exclusion was required to eliminate confounding factors that are difficult to standardize, it should be noted that patients requiring NCCL restorations generally also suffer from those conditions. Additionally, 24 months is not long enough to attain conclusive outcomes; therefore, the current study requires further follow-ups. Likewise, other clinical trials comparing “no wait” universal adhesives with different self-etch and etch-and-rinse adhesives under more challenging oral conditions should be conducted, with the patients followed long-term to determine the real-life clinical performance of simplified adhesives.

## CONCLUSION

The “no wait” universal adhesive (Clearfil Universal Bond Quick) tested in this study exhibited good clinical performance in NCCL restorations after 24 months when etch-and-rinse and selective enamel-etching strategies were used, as with the other etch-and-rinse adhesive (Tetric N-Bond Universal) tested. Restorations using the self-etch adhesive (Clearfil SE Bond) and the “no wait” universal adhesive, applied in a self-etch bonding mode, were associated with lower survival rates and exhibited significantly more deterioration in terms of marginal adaptation and discoloration compared to the other groups at the 24-month follow-up.

## Clinical relevance:

The “no wait” universal adhesive showed acceptable clinical performance when applied in selective etch and etch-and-rinse mode, but higher failure rates were observed with the adhesive applied in self-etch mode.
